# BT595, a 10% Human Normal Immunoglobulin, for Replacement Therapy of Primary Immunodeficiency Disease: Results of a Subcohort Analysis in Children

**DOI:** 10.1007/s10875-022-01397-0

**Published:** 2022-11-16

**Authors:** Gergely Kriván, Michael Borte, Pere Soler-Palacin, Joseph A. Church, Ildiko Csurke, James B. Harris, Jay A. Lieberman, Isaac R. Melamed, James N. Moy, Reka Simon, Silke Aigner, Stephan Lentze, Christiane Staiger

**Affiliations:** 1 Department of Pediatric Hematology and Stem Cell Transplantation, United St. Istvan and St Laszlo Hospital, Albert Florian u. 5-7, Budapest, Hungary; 2 ImmunoDeficiency Center Leipzig (IDCL) at Klinikum St. Georg gGmbH, Leipzig, Germany; 3 Children’s Hospital, Vall d’Hebron Barcelona Hospital Campus, Barcelona, Catalonia Spain; 4 Children’s Hospital Los Angeles, Los Angeles, CA USA; 5 Szabolcs-Szatmar-Bereg Megyei Korhazak és Egyetemi Oktatokorhaz, Nyíregyháza, Hungary; 6 The South Bend Clinic, South Bend, IN USA; 7 LeBonheur Children’s Hospital, Memphis, TN USA; 8 IMMUNOe Research Centers, Centennial, CO USA; 9 Rush University Medical Center, Chicago, IL USA; 10 Borsod-Abauj-Zemplen Megyei Korhaz és Egyetemi Oktato Korhaz, Miskolc, Hungary; 11 Biotest AG, Landsteinerstr. 5, Dreieich, Germany

**Keywords:** Children, Primary immunodeficiency (diseases) (PID), Intravenous immunoglobulin (IVIg), Serious bacterial infections, Clinical trial, Safety, Tolerability, Efficacy, Pharmacokinetics

## Abstract

**Purpose:**

To assess the efficacy, pharmacokinetics, and safety of a new, highly purified 10% IVIg (BT595, Yimmugo^®^) administered in children with PID.

**Methods:**

This was an open-label, prospective, uncontrolled, multicenter Phase III pivotal trial. Among the 67 subjects in the trial were 18 pediatric patients aged 2 to 17 years with diagnosis of PID included in this analysis. They received doses between 0.2 and 0.8 g/kg body weight for approximately 12 months at intervals of either 3 or 4 weeks. Dosage and dosing interval were based on each patient’s pre-trial infusion schedule. The rates of acute serious bacterial infections (SBI), secondary efficacy, safety, and pharmacokinetic outcomes were evaluated.

**Results:**

No SBI occurred in the pediatric population. Two hundred sixty infusions were administered to the 18 pediatric patients. The mean (SD) IgG trough level was 8.55 (1.67) g/L at baseline and 8.84 (2.17) g/L at the follow-up visit after the last BT595 infusion. At the single infusions respectively, the average mean IgG trough levels ranged between 8.52 and 10.58 g/L. More than 85% of all infusions administered were not associated with any infusional AE (start during or within 72 h post-infusion). None of the severe or serious AEs were related to the investigational medicinal product (IMP). No premedication was used. Thirteen children reached a maximum infusion rate between > 2.0 and 8 mL/kg/h; no AE with an onset during the infusion occurred at these infusion rates.

**Conclusion:**

BT595 is effective, convenient, well tolerated, and safe for the treatment of children with PID.

**Trial registration:**

EudraCT: 2015–003652-52; NCT02810444, registered June 23, 2016.

## Introduction

Primary immunodeficiency diseases (PID)/inborn errors of immunity (IEI) are a class of about 430 disorders characterized by an intrinsic defect in the human immune system [1]. Patients with antibody deficiency, including children, present with recurrent, often severe, bacterial infections more than usually observed for their age group [2]. These infections affect the respiratory system, GI tract, and other organs [3]. Such infections associated with PID, its negative sequelae, and complications predispose for autoimmunity and malignancies, and are associated with increased morbidity and mortality. After infection, malignancy is the most prevalent cause of death [4]. In PID, early diagnosis and immunoglobulin replacement therapy (IGRT) is important [5].

BT595 (Yimmugo^®^, a registered trademark in the EU and Switzerland; in the USA, the trademark application is pending; Biotest AG, Dreieich, Germany) is a novel, highly purified 10% preparation of human normal immunoglobulin for intravenous administration (IVIg). The product has been investigated for replacement therapy in PID in a clinical trial with 67 patients, including both children and adults [6]. Here, we report the results for the pediatric subgroup of this trial.

## Methods

The clinical trial protocol and all other relevant documents were reviewed and approved by the competent independent ethics committees before the trial commenced. The trial was conducted in accordance with the International Council for Harmonization of Technical Requirements for Pharmaceuticals for Human Use (ICH) Good Clinical Practice (GCP) guidelines, and the Declaration of Helsinki.

For minors who could have only been enrolled in the trial with the consent of their parent(s) or legally acceptable representative, the minor patients were informed about the trial commensurate with their understanding, which was also documented.

### Study Product

BT595 was manufactured by using plasma from healthy donors. The final, sugar-free, and glycine-stabilized product contains 100 g human plasma protein per liter (i.e., 10% solution). We report here the pediatric partial results of a trial investigating the long-term, low-dose IgG replacement therapy in patients with PID.

### Study Design

This was an open-label, prospective, uncontrolled, multicenter Phase III pivotal trial to assess the efficacy, safety, and pharmacokinetics (PK) of BT595 in pediatric and adult patients with PID (EudraCT: 2015–003652-52; NCT02810444). Details on the trial design and eligibility criteria have been published previously [6].

The primary objective was to demonstrate that the rate of acute serious bacterial infections (i.e., the mean number of acute serious bacterial infections [SBIs] per subject-year) was less than 1.0, to provide substantial evidence of efficacy. Specific diagnostic criteria for SBIs were used as per the Food and Drug Administration (FDA) guidance and included any AE leading to the following Medical Dictionary for Regulatory Activities (MedDRA) preferred terms (PTs): sepsis, bacterial sepsis, bacteremia, meningitis bacterial, osteomyelitis bacterial, arthritis bacterial, pneumonia bacterial, and abdominal abscess [7]. Furthermore, all AEs leading to any other PT were reviewed by the sponsor for a possible relationship to the primary endpoint (e.g., AEs including one of the following words: bacteremia, sepsis, meningitis, osteomyelitis, arthritis, pneumonia, and abscess). Identified AEs possibly related to the primary endpoint were queried to the investigator to clarify if the AE fulfilled the FDA-defined SBI criteria. Any SBI had to be confirmed by objective findings (e.g., X-ray, laboratory data). The secondary objectives of this trial, in addition to further efficacy assessments, were to assess the safety and pharmacokinetic (PK) characteristics of BT595. In addition, health-related quality of life exploratory endpoints [8] were evaluated.

### Subjects and Treatment

Pediatric male or female subjects aged 2 years or above were eligible for participation if they had a diagnosis of PID with impaired antibody production, i.e., common variable immunodeficiency (CVID) or X-linked agammaglobulinemia (XLA), as defined by the diagnostic criteria of the European Society for Immunodeficiencies (ESID) and the Pan American Group for Immunodeficiency (PAGID) [9, 10].

After an initial, up to 28-day screening period, patients were switched from their pre-trial IVIg replacement therapy to BT595 at the baseline visit. Patients were assigned to receive BT595 at doses between 0.2 and 0.8 g per kg body weight (bw) (2 to 8 mL/kg bw), either at a Q3W or Q4W schedule, for a treatment period of approximately 12 months. The dose and dosage interval had to be consistent with the subject’s pre-trial IVIg dose and was only to be changed if medically indicated. Efficacy and safety were assessed from baseline (week 0) to the closing (follow-up) visit at week 54 (Q3W schedule) or week 56 (Q4W schedule).

For the first infusion, BT595 was administered as intravenous infusion at an initial infusion rate of 0.3 mL/kg/h for 30 min, to be increased to 1.4 mL/kg/h for a further 30 min. If well tolerated, the infusion rate could then be gradually increased to a maximum of 2 mL/kg/h for the remainder of the infusion. From the subsequent infusion onwards, subjects’ infusion rates could be gradually increased to a maximum of 8 mL/kg/h at the investigator’s discretion, following the initial infusion rate of 0.3 mL/kg.

### Pharmacokinetics

The number of blood draws depended on the patient’s age category. Blood samples were collected with procedures adapted for each age category (e.g., pediatric tubes and plasma micro-sampling). A homecare service was allowed for the PK sampling of pediatric patients.

Serum IgG trough levels (total IgG) were determined in all patients before each administration. In addition, serum samples for analysis of PK parameters at steady state (*C*_max_, *t*_max_, AUC_tau_, CL_ss_, *t*_1/2_) were taken pre-dose before infusion 7 (Q3W schedule) or infusion 5 (Q4W schedule), and at a series of fixed time points after this infusion according to their age category. Time points for patients ≥ 6 years were as follows: pre-dose (10 to 30 min before infusion), 10 to 30 min post-infusion, 4 and 24 h post-infusion; and 4, 7, 14, and 21 days (for the Q3W and Q4W schedule), and 28 days post-infusion (Q4W schedule only).

For children of 2 to < 6 years, optional sparse PK sampling at flexible time points within specified time windows after the end of the infusion was conducted.

The blood samples taken for PK analysis were analyzed by a central laboratory using standard methodology.

### Statistical Analysis

The statistical analysis was performed using SAS^®^ version 9.4. The safety set (SAF) included all subjects who received at least one dose of study medication and was used for safety evaluation. The full analysis set (FAS) was used for efficacy evaluation and was identical to the SAF. Steady-state PK parameters were derived from a non-compartmental analysis, using the data from dense PK sampling.

The minimum sample size of 50 evaluable subjects, including at least 20 pediatric subjects, followed the EMA guidelines [11] and the BT595 pediatric investigational plan (PIP). This ensured a power of at least 80% to reject the null hypothesis of ≥ 1 SBI/subject/year. A one-sided one-sample Poisson test with type I error of 0.01 was chosen, assuming a true underlying SBI rate of 0.5 per subject/year.

The proportion of infusions associated with ≥ 1 infusional AE (start during or within 72 h post-infusion) was calculated and the exact upper one-sided 95% confidence interval (CI) limit was compared to the threshold of 0.40 specified in the FDA Guidelines [12].

### Data and Safety Monitoring Board (DSMB)

To monitor the safety data and provide advice and recommendations on the enrollment of pediatric patients, a DSMB consisting of independent experts was convened. The pediatric cohort was opened for enrollment after acceptable safety and tolerability had been demonstrated in ≥ 10 adult patients (18 to < 76 years) who had received ≥ 2 BT595 infusions with no safety concerns.

## Results

### Subject Demographics

Of the 67 subjects eligible for the trial and treated, 18 (26.9%) were pediatric patients (2 to 16 years). Adolescents were defined as 12 to < 17 years according to the FDA guidance [13] and 12 to < 18 years according to the EMA guidance [14]. Since the only patient aged 17 years at screening (initial visit) withdrew consent prior to the first BT595 infusion, the number of subjects in the FAS qualifying as adolescents and adults comply with both FDA and EMA age group categorizations. Details of patient characteristics are given in Table 1.

**Table 1:** Demographics characteristics of pediatric subjects

Characteristics	Q3W (*N* = 4)	Q4W (*N* = 14)	Total (*N* = 18)
Gender (*n*, %)			
Male	3 (75.0)	12 (85.7)	15 (83.3)
Female	1 (25.0)	2 (14.3)	3 (16.7)
Age at screening (years)			
Mean (SD)	7.5 (4.20)	10.2 (4.08)	9.6 (4.15)
Median	7.0	11.0	10.5
Minimum, maximum	3–13	2–16	2–16
Age group (*n*)			
2 to < 6 years	1	2	3
6 to < 12 years	2	7	9
12 to < 17/18 years	1	5	6
Race (*n*)			
Caucasian	4 (100)	13 (92.9)	17 (94.4)
Asian	0	1 (7.1)	1 (5.6)
Height (cm)			
Mean (SD)	130.10 (29.509)	146.49 (25.843)	142.84 (26.711)
Median	128.50	156.70	151.50
Minimum, maximum	95.8–167.6	90.0–178.3	90.0–178.3
Weight (kg)			
Mean (SD)	30.78 (14.082)	49.01 (22.295)	44.96 (21.817)
Median	29.25	54.45	44.85
Minimum, maximum	15.5–49.1	12.5–85.1	12.5–85.1
BMI (kg/m^2^)			
Mean (SD)	17.52 (2.443)	21.39 (5.228)	20.53 (4.969)
Median	17.18	21.76	19.81
Minimum, maximum	14.9–20.8	13.3–29.6	13.3–29.6

*N*, number of pediatric subjects; *n*, number of subjects in a specified category; *Q3W*, 3-week schedule; *Q4W*, 4-week schedule; *SD*, standard deviation

Adolescents were defined as 12 to < 17 years according to the FDA guidance and 12 to < 18 years according to the EMA guidance. Since the only subject aged 17 years at screening (initial visit) withdrew consent prior to the first BT595 infusion, the number of subjects in the FAS qualifying as adolescents and adults comply with both FDA and EMA age group categorizations

Eighteen out of the planned 20 pediatric patients were enrolled, despite significant efforts to identify more participants for the trial. Furthermore, only three children were enrolled in the 2 to < 6 year age group. Notably, in the joint age group 2 to < 12 years, 12 patients were treated with BT595. The main reasons given for non-participation in the trial were refusal to consent, blood sampling amount, and not being on established IVIg therapy and/or stable IVIg dose. The efforts taken by the sponsor to boost the number of pediatric patients included protocol amendments to facilitate pediatric recruitment, the opening of additional trial sites, motivational visits to existing sites, and in total three prolongations of the children recruitment period.

The pediatric patients were predominantly male (15 patients [83.3%]), and were treated at 10 sites in 4 countries: in the USA 5 patients, 8 in Hungary, 3 in Germany, and 2 in Spain. The majority of children were White, and there was a single 11-year-old Asian boy who was treated in the USA.

Ten patients were diagnosed with XLA and 7 patients with CVID (Table 2). Especially, in the joint age group 2 to < 12 years, XLA was the dominant PID diagnosis, while in the age group 12 to < 17 years, CVID predominated. One additional patient had another specific antibody defect, and was allowed to participate in the trial, but was excluded from the per protocol set (PPS). A 13-year-old girl in the Q3W schedule group received several different antibiotics from the start of the trial onwards and for up to 189 days as treatment for a pre-existing Lyme disease, and was excluded also from the PPS. A 3-year-old boy discontinued after 6 cycles early because the subject’s guardian withdrew consent.

**Table 2:** Disease characteristics at baseline—pediatric subjects (*N* = 18)

Criterion	2 to < 6 y (*N* = 3)	6 to < 12 y (*N* = 9)	12 to < 17/18 y (*N* = 6)	Total (*N* = 18)
Type of diagnosis (*n*)				
CVID	0	2	5	7
XLA	3	6	1	10
Other	0	1	0	1
Time since diagnosis (months)				
Mean (SD)	17.3 (11.55)	75.4 (42.64)	58.2 (51.79)	60.0 (45.92)
Median	24.0	80.0	52.0	57.5
Minimum, maximum	4–24	6–137	9–136	4–137
Total IgG trough level at baseline (g/L)				
Mean (SD)	8.49 (0.85)	7.93 (1.46)	9.50 (2.01)	8.55 (1.67)
Median	8.10	7.78	9.91	8.38
Minimum, maximum	7.9–9.5	5.3–10.7	6.0–11.4	5.3–11.4

*CVID*, common variable immunodeficiency; *N*, number of subjects; *n*, number of subjects in a specified category; *SD*, standard deviation; *XLA*, X-linked agammaglobulinemia

### BT595 Treatment

Two hundred sixty infusions with a total of 5257 g (52,566 mL) were administered to the 18 pediatric patients (Table 3). The subject-years of BT595 exposure of the different age groups (2 to < 6 years, 6 to < 12 years, and 12 to < 17/18 years) were 2.61, 9.85, and 6.49 subject-years, respectively. The BT595 total dose administered per patient differed between age groups due to the differences in body weight. The mean (SD) actual dose administered per infusion was 0.42 (0.09) g/kg bw (range 0.27-0.68).

**Table 3:** Summary of exposure to BT595 by age group (*N* = 18)

Criterion	2 to < 6 y (*N* = 3)	6 to < 12 y (*N* = 9)	12 to < 17/18 y (*N* = 6)	Total (*N* = 18)
Duration of exposure (days)				
Mean (SD)	287.3 (157.58)	372.0 (11.66)	368.7 (12.94)	356.8 (63.70)
Median	365	365	365	365
Minimum, maximum	106–391	364–393	358–393	106–393
Total number of infusions	35	136	89	260
Mean (SD)	11.7 (4.93)	15.1 (1.69)	14.8 (1.60)	14.4 (2.57)
Median	14	14	14	14
Minimum, maximum	6–15	14–18	14–18	6–18
Total dose across all infusions (g)	263	2576	2417	5257
Mean (SD)	7.7 (2.16)	19.3 (6.56)	26.9 (8.43)	19.9 (9.25)
Median	8	19.3	27.5	19.6
Minimum, maximum	5.36–9.64	11.95–31.57	15.0–36.22	5.36–36.22
Actual calculated dose across all infusions (g/kg bw)				
Mean (SD)	0.39 (0.10)	0.43 (0.06)	0.42 (0.14)	0.42 (0.09)
Median	0.39	0.43	0.38	0.41
Minimum, maximum	0.29–0.49	0.34–0.53	0.27–0.68	0.27–0.68

*bw*, body weight; *N*, number of subjects; *SD*, standard deviation; *y*, years

### Primary Endpoints

None of the 18 pediatric patients experienced an SBI.

### Secondary Endpoints

The mean and median total IgG trough levels remained nearly constant throughout the trial in both schedule groups. The average IgG trough levels remained well above the targeted minimal trough level of 5 g/L. In the overall pediatric population, the mean (SD) trough level was 8.55 (1.67) g/L at baseline and 8.84 (2.17) g/L at the follow-up visit after the last BT595 infusion. Corresponding median values were 8.4 g/L at baseline and 8.3 g/L at the follow-up visit. At the single infusions respectively, the average mean IgG trough levels ranged between 8.52 and 10.58 g/L. Of all 18 pediatric patients, only 1 child in the 6 to < 12 years age group had a single trough level of < 5 g/L (4.71 g/L, before infusion 7).

No pediatric patient was withdrawn due to infection. A high-level summary of secondary endpoints is given in Table 4. Eleven children needed ≥ 1 antibiotic treatment.

**Table 4:** High-level summary of secondary endpoints and maximum infusion rates—pediatric subjects (*N* = 18)

Parameter	2 to < 6 y (*N* = 3)	6 to < 12 y (*N* = 9)	12 to < 17/18 y (*N* = 6)	Total (*N* = 18)
Total IgG trough levels before each infusion, mean (SD)				
Baseline (before 1st inf.)	8.49 (0.849)	7.93 (1.456)	9.50 (2.006)	8.55 (1.671)
Steady state (before 5th inf.)	9.66 (1.363)	8.48* (0.986)	9.46 (1.796)	9.03 (1.401)
Rate of non-serious infections				
* n* (%) with ≥ 1 infection	3 (100)	6 (66.7)	6 (100)	15 (83.3)
Rate per subject-year^a^	6.90	3.15	3.08	3.64
Antibiotic treatment (includes prophylactic treatment)				
* n* (%) with antibiotic treatment	3 (100)	4 (44.4)	4 (66.7)	11 ( 61.1)
Days per subject-year^b^	18.02	55.93	36.04	43.90
Rate of time lost from school/work due to infections and their treatment				
* n* (%) with any time lost (≥ 1 day)	2 (66.7)	6 (66.7)	4 (66.7)	12 (66.7)
Hospitalizations due to infections				
* n* (%) with hospitalization	1 (33.3)	0	0	1 (5.6)
Days per subject-year^b^	7.67	0.00	0.00	1.06
Fever episodes				
* n* (%) with fever episodes	1 (33.3)	4 (44.4)	3 (50.0)	8 (44.4)
Days per subject-year^b^	7.28	1.42	3.85	3.06
Maximal administered infusion rate in mL/kg/h, *n* (%)				
≤ 0.3	0	0	0	0
> 0.3 to ≤ 1.4	1 (33)	1 (11.1)	0	2 (11.1)
> 1.4 to ≤ 2	1 (33)	1 (11.1)	1 (16.7)	3 (16.7)
> 2 to ≤ 4	1 (33)	3 (33.3)	2 (33.3)	6 (33.3)
> 4 to ≤ 6	0	2 (22.2)	2 (33.3)	4 (22.2)
> 6	0	2 (22.2)	1 (16.7)	3 (16.7)
Thereof 8	0	2 (22.2)	1 (16.7)	3 (16.7)

*CI*, confidence interval; *d*, day; *IgG*, immunoglobulin G; *inf*., infusion; *N*, number of subjects; *n*, number of subjects in a specified category; *SD*, standard deviation; *y*, year

^*^n = 8

^a^Total number of events divided by total number of subject-years

^b^Total number of days with events divided by the total number of subject-years

Infections (defined by MedDRA SOC Infections and Infestations) that were reported as treatment-emergent adverse events (TEAEs, i.e., occurred after first IMP administration) at least once by ≥ 2 pediatric patients were nasopharyngitis (5 patients [27.8%], 9 events), upper respiratory tract infections (5 patients [27.8%], 12 events), viral upper respiratory tract infection (5 patients [27.8%], 5 events), bronchitis (3 patients [16.7%], 5 events), and conjunctivitis, influenza, urinary tract infection, and viral infection (each: 2 patients [11.1%], 2 events). Apart from 2 infections in a 3-year-old subject with XLA described below, all infections were mild or moderate in severity and non-serious TEAEs.

A 3-year-old boy with XLA had a total of 18 infections of various types, including 1 non-treatment-emergent infection, most of them were typical pediatric infections. Two of these were serious adverse events (SAEs) and required hospitalization (appendicitis and gastrointestinal viral infection). Seven of these infections required antibiotic therapy—3 bronchitis events, 1 otitis media, 1 pharyngitis, 1 conjunctivitis, and 1 appendicitis—including parenteral antibiotics for the events of appendicitis and otitis media. Additionally, he was hospitalized for a third SAE, which was a moderate dehydration, due to infection.

An 11-year-old boy with XLA had 10 treatment-emergent infections of various types, including 3 ulcerations, 2 pyrexia, and 1 chest cold/cough. Two staphylococcal skin infections (lower trunk) required local antibiotic therapy. In addition, the child received prophylactic antibiotic treatment with azithromycin throughout the trial (409 days) to prevent infections due to his XLA. In addition, the patient received mupirocin for the treatment of a staphylococcal skin infection for an imputed duration of 342 days. The imputation was made due to an incomplete stop date, applying a worst case approach.

Furthermore, 3 of the 9 patients in the 6 to < 12 years age group, all with XLA, had 10, 6, and 5 infections, respectively. Two of the 6 patients in the 12 to < 17/18 years age group with CVID had 8 and 5 infections, respectively.

Mainly due to these patients, the annual rate of any treatment-emergent infections was higher in the 3 pediatric age groups than in adults or in the overall population. The rate was highest in the small group of children aged 2 to < 6 years (7.67) compared the overall pediatric population aged 2 to < 17 years (3.75) and adults (2.43). Fever was reported in 8 pediatric patients.

The assessment for time lost from school/work included in the children’s age groups also absence from other childcare option, e.g., kindergarten. The proportion of patients with any time lost from school/work (66.7%) was identical in all 3 pediatric age groups and considerably higher than in adults (28.6%). The apparent high annual rate of 33.74 days per subject-year (365 days) in the small group of 3 young children (2 to < 6 years) was caused by the single 3-year-old White male patient already mentioned above who lost 85 days; the 2 other children of this age group lost no days and < 7 days, respectively.

A number of methods for the assessment of health-related quality of life were analyzed as exploratory endpoints in this trial. The PedsQL™ Measurement Model for the Pediatric Quality of Life Inventory™ version 4.0 (PedsQL™; child self-report and/or parent proxy report) was evaluated using the respective tools for different pediatric age groups (2 through 4 years, 5 through 7 years, 8 through 12 years, and 13 through 18 years). The mean (SD) PedsQL™ total score increased from 91.7 (14.43) at baseline to 94.2 (8.14) at the last protocol-defined infusion (infusion 18) in the Q3W schedule group and from 81.0 (10.65) at baseline to 86.4 (12.05) at infusion 14 in the Q4W schedule group. In an assessment for the feasibility, reliability, and validity of PedsQL™, the minimal clinically important difference of a score change of 4.4 for the total child self-reported score has been proposed [15]. Published normative data for the PedsQL™ total score identified a mean score of 82.7 for a healthy population from the USA [16].

For the EQ-5D-Y™, most of the 15 pediatric patients (4 to 17 years, inclusive) at baseline and all pediatric patients with data available at the time of the last protocol-defined infusion reported no problems at all for all 5 dimensions, with no clinically relevant differences between the various age groups. Correspondingly, the pediatric patients also had high ratings of their overall health state (EQ VAS) throughout the trial. Mean (SD) EQ VAS ratings for the pediatric patients of both schedule groups combined were 90.3 (7.83) at baseline, increased to 95.4 (6.31) at infusion 2, and then remained in the range between 91.7 and 96.5 up to the last protocol-defined infusion.

### Safety

There were no fatal or life-threatening AEs; no subject was withdrawn due to AE from the trial and no patient required any rescue medication.

Overall, 18 children experienced a total of 184 TEAEs, which included the infections as well. The majority of these TEAEs were non-serious AEs (180 events, 97.8%) of mild (148 events, 80.4%) or moderate (34 events, 18.5%) severity and not related to study medication. Two patients, both 3-year-old boys with XLA, experienced a total of 2 severe TEAEs, both non-infusional, serious adverse event (SAEs), which were not related to study medication. They comprised an SAE of severe appendicitis in one child and a SAE of severe thermal burn in the other boy who touched a hot oven. Of note, a cluster of 28 events of epistaxis was reported by 3 pediatric patients (4.5%).

Two of the 18 children (11.1%) experienced 4 non-infusional SAEs that were not related to study medication. These were the abovementioned 3-year-old boys mentioned in the previous paragraph. The 4 SAEs comprised the severe appendicitis described above plus 2 SAEs of moderate severity, gastrointestinal viral infection, and dehydration in the same boy, and the thermal burn mentioned above.

None of the severe or serious AEs in pediatric subjects were related to study medication or occurred during or within 72 h after infusion of the study drug.

In total, 9 children (50.0%) experienced 12 TEAEs that were assessed as related to study medication (adverse drug reactions [ADRs]). All ADRs were infusional AEs of mild or moderate severity. The 12 ADRs include 4 infusional AEs of “extra dose administered” in 4 patients, who received an additional final infusion in violation of the protocol.

The only other ADR observed more than once in the pediatric population was headache (4 patients [22.2%], 4 events). The 3 children aged 2 to < 6 years had no ADR with the exception of one event of extra dose administration.

In each of the different pediatric age groups assessed, > 85% of all infusions administered were not associated with any TEAEs. The proportion of infusions temporally associated with ≥ 1 (infusional) AE was 0.14 events per infusion. In 18 children, 28 of 260 infusions (10.8% [upper limit of the 1-sided 95% CI: 14.5%]) were associated with infusional AEs, with no meaningful differences between the pediatric age groups (Table 5). This was well below the FDA-required safety threshold of 0.40 overall and for each age group. Overall, 15 of the 18 pediatric patients (83.3% [90% CI: 62.3 to 95.3]) reported a total of 36 infusional AEs, all were non-serious and of mild or moderate severity. Infusional AEs, apart from of extra dose administration, observed more than once in the pediatric population were headache (7 patients [38.9%%], 10 events), fatigue (2 patients [11.1%], 2 events), epistaxis (1 patient [5.6%], 2 events), and oropharyngeal pain (1 patient [5.6%], 2 events).

**Table 5:** Summary of infusions temporally associated with TEAEs—pediatric subjects (*N* = 18**)**

Parameter	2 to < 6 y (*N* = 3)	6 to < 12 y (*N* = 9)	12 to < 17/18 y (*N* = 6)	Total (*N* = 18)
Total number of infusions administered, *n*	35	136	89	260
Infusions not associated with any TEAE, *n* (%)	31 (88.6)	123 (90.4)	78 (87.6)	232 (89.2)
Infusions associated with ≥ 1 TEAE, *n* (%) (upper limit of the 1-sided 95% CI)	4 (11.4) [24.3]	13 (9.6) [14.8]	11 (12.4) [19.6]	28 (10.8) [14.5]
Mean number of infusional AEs per infusion, *n*	0.14	0.11	0.18	0.14

*N*, number of subjects; *n*, number in a specified category; *y*, years; *CI*, confidence interval; *TEAE*, treatment-emergent adverse event; *infusional AE*, AE start during or within 72 h post-infusion

The maximum infusion rate of 8 mL/kg/h was used in 3 of the 18 pediatric patients (16.7%), additional 4 reached an infusion rate of > 4.0 mL/kg/h, and all of them were in the age groups 6 to < 12 and 12 to < 17/18 years (Table 4). None of the AEs with an onset during the infusion occurred at infusion rates of > 2.0 mL/kg/h.

In this trial, no premedication was used in the pediatric population.

### Pharmacokinetics

**Fig. 1:**
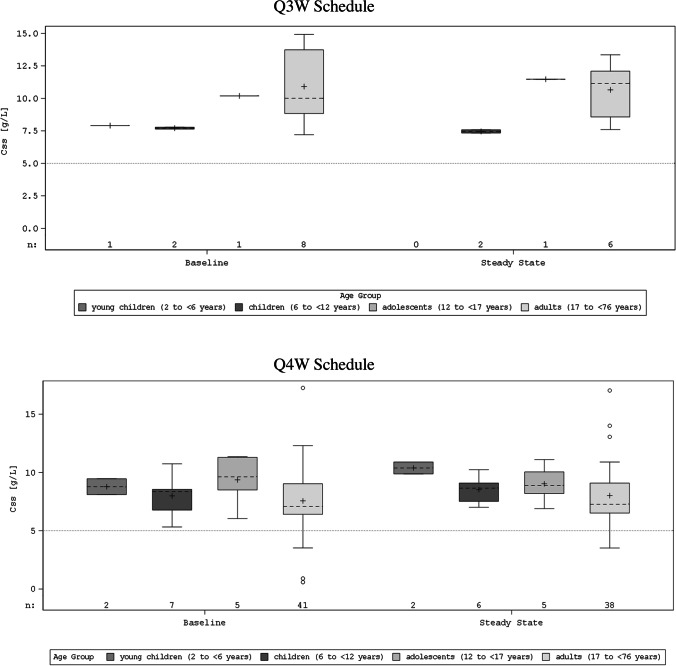
Trough levels of total IgG (box plots) at steady state for BT595 and previous immunoglobulin by age group—PK trough set (*N* = 67). Css, concentration at steady state; IgG, immunoglobulin G, IQR, interquartile range (25 to 75% percentile); IVIg, immunoglobulin for intravenous administration; Min, minimum; Max, maximum; Q3W, 3-week schedule; Q4W, 4-week schedule. The cross symbol represents arithmetic mean and the dashed line represents the median value. The box represents the 1st and 3rd quartile (25th and 75th percentile). The whiskers represent the Min–Max range, or the 1.5-fold IQR in case of outliers (beyond the 1.5-fold IQR, shown as circles). Note: Steady-state concentrations of total IgG were assessed at local laboratories at baseline (steady state for reference therapy; after ≥ 3 months on the same IVIg reference treatment) and before infusion 7 or infusion 5 of BT595 (for the Q3W and Q4W schedules, respectively). Note: Horizontal dotted line refers to the targeted minimal trough level of 5 g/L

The total IgG trough levels at steady state (local laboratory assessments) for the different age groups (2 to < 6 years, 6 to < 12 years, 12 to < 17/18 years, 17/18 to < 76 years) are presented in Fig. 1. Trough levels (*C*_ss_) did not differ notably between BT595 and patients’ previous IVIg reference therapy as seen by comparison between baseline and steady state. Further pharmacokinetic parameters of the Q3W and Q4W schedules are given in Table 6.

**Table 6:** Pharmacokinetic parameters—pediatric subjects (6 to < 17 years), dense PK subset, all doses

Parameter	Mean	SD	Median	Minimum	Maximum
Q3W (*N* = 3)					
* C*_max_ (g/L)	24.7	9.2	22.4	16.9	34.8
* t*_max_ (day)	na	na	0.27	0.11	0.28
AUC_tau_ (day*g/L)	318.1	137.6	242.9	235.0	477.0
CL_ss_ (L/day)	0.063	0.001	0.063	0.062	0.064
* t*_1/2_ (day), (*N* = 2)	na	na	22.5	15.5	29.5
Q4W (*N* = 6)					
* C*_max_ (g/L)	23.04	2.3	23.9	19.41	25.54
* t*_max_ (day)	na	na	0.24	0.17	4.23
AUC_tau_ (day*g/L)	407.4	75.0	392.1	316.0	524.0
CL_ss_ (L/day)	0.057	0.016	0.057	0.038	0.079
* t*_1/2_ (day) (*N* = 3)	40.3	15.1	33.8	29.6	57.5

*N*, number of observations contributing to statistic; *na*, not available; *Q3W*, 3-week schedule; *Q4W*, 4-week schedule; *SD*, standard deviation

## Discussion

This trial provides results from 18 patients aged 2 to 16 years treated with at least one infusion of BT595. Seven patients were diagnosed with CVID, mainly older children, 10 patients had XLA, and one additional child had a specific other antibody defect. The children were predominantly male (15 patients [83.3%]) due to the high number of subjects with XLA.

Overall, the efficacy results demonstrate that BT595 is effective as replacement therapy in pediatric PID patients. No pediatric patient experienced an SBI. The outcomes of the secondary efficacy endpoints were consistent with the product being effective, and supported the efficacy of BT595 in all pediatric age groups and were comparable to adults.

The rate of treatment-emergent infections was highest in the small group of children aged 2 to < 6 years (7.67) compared the overall pediatric population aged 2 to < 17 years (3.75). This was expected, as pediatric subjects have more infections due to their environment in childhood. Moreover, the subgroup of the young children aged 2 to < 6 years included only patients with XLA. All severe TEAEs and all SAEs occurred in this age group. Within this group was a 3-year-old boy with multiple infections and AEs, both of long duration. This impacted the results of this small subgroup considerably. The 3 patients of this age group had no ADRs other than one event of extra dose administration.

Compared with other pediatric PID studies of IVIg previously described in literature, we had a strikingly high proportion of children with XLA (Table 7). Patients with XLA have a deficient development of B-lymphocytes, whereas in patients with CVID, a T-cell deficit is more prominent [17], resulting in agammaglobulinemia versus hypogammaglobulinemia, respectively. Children with XLA often suffer from more infections like, e.g., otitis, sinusitis, pneumonia, chronic/recurrent diarrhea, conjunctivitis, meningitis/encephalitis, sepsis, and septic arthritis more often and more severely [17]. Because the population in our trial included more children with XLA in particular in the two younger age groups, certain infection-related secondary endpoints appeared to be worse compared to other studies (Table 7). However, our trial demonstrated that SBIs could also be reliably prevented in children with XLA. In addition, the typical infections seen in pediatric patients with XLA [17, 18] had a low frequency or were not reported at all in our pediatric subgroup.

**Table 7:** Comparison with other studies for IGRT in PID pediatric patients (comparison to literature)

Criterion	BT595	Ballow et al. 2016 [17]	Ochs et al. 2018 [3] (NGAM-01)	Melamed et al. 2016 [18]
Number of subjects, *n*	18	24	25	25
Children with XLA, *n* (%)	10 (55.6)	7 (29.2)	5 (20)	3 (12)
Subjects with SBI, *n* (%)	0	1 (4.2)	1 (4)	2 (8)
Subjects with other/non-SBI infections, *n* (%)	15 (83.3)	9 (37.5)	22 (88)	21 (84)
Subjects who experienced at least one ADR, *n* (%)	9 (50)	20 (83.3)	5 (20)	14 (56)
Total ADR number, *n*	12, incl. 4 extra dose administered	159	na	na
Subjects with most common ADRs, as defined in respective source, *n* (%)	Each 4 (22.2): extra dose administered, headache	Headache 10 (41.7) Pyrexia 7 (29.2) Hypotension 6 (25.0) Tachycardia 6 (25.0) Diastolic hypotension 5 (20.8)	Headache 2 (8.0) Chills 2 (8.0) Abdominal pain 2 (8.0)	AR* in 3 subjects: Headache 11 (44.0) Sinusitis 6 (24.0) Hypotension 4 (16.0) Each 3 (12): Tachycardia, pyrexia, hypertension, fatigue, infusion-site reaction, dry skin, nasal congestion, upper respiratory tract infection, abdominal pain

*ADR*, adverse drug reaction; *AR*, adverse reaction; *N*, number of subjects; *n*, number in a specified category; *na*, not available; *SBI*, acute serious bacterial infection; *XLA*, X-linked agammaglobulinemia

^*^Adverse reaction (AR) was defined as a treatment-emergent adverse event that began during an infusion or within 72 h after completion of an infusion, which was considered by investigators to be possibly, probably, or definitely related to study drug, or for which the investigator’s causality assessment was either missing or indeterminate

Ballow et al. reported an upper CI of ≤ 30.3% for the unadjusted percent of infusions with a non-treatment-related infusional AE [19]. In the study of Melamed et al., 97 (26.4%) of the 368 total infusions were associated with an AE up to 72 h after the infusion (regardless of causality) [20] with an upper 95% CI of 0.304 (30.4%). In contrast, the upper limit of the 1-sided 95% CI in pediatric patients was only 14.5% in our trial. Twenty-eight of 260 infusions (10.8%) were associated with infusional AEs without meaningful differences between the age groups.

The rate of ADRs was equivalent or significantly lower compared with other pediatric trials (Table 7). Headache was the only ADR observed with a frequency of > 5%, beside the extra dose administration, which was unintentionally but documented as an AE.

Seven of the 18 children achieved maximum infusion rates of > 4.0 mL/kg/h, 3 of them 8 mL/kg/h (Table 4). These were well tolerated, with no AE occurring during infusion. This illustrates that an individualized approach to high infusion rates is also the tool of choice in pediatric patients. No pediatric patient required a premedication. Although practice varies among countries, patients are often premedicated with antihistamines, antipyretics, and/or steroids to avoid adverse events (AEs) occurring in conjunction with the infusion of IVIg products.

The PK data of BT595 are in line with the well-characterized PK profile of other licensed IVIg products showing a long terminal half-life (> 20 days) and a low clearance. Although the number of pediatric subjects in the PK subset was low (in total 9 subjects), PK of IgG in children and adults can be considered similar [21].

This is not the first IVIg trial that struggled to enroll (very) young subjects resulting in delays and increased costs. However, our results confirm clinical experience that IVIg have equal properties and effects in children and adults. Given the difficulties in recruitment, it should be reconsidered whether a minimum number of children are really necessary and whether regulatory requirements may be unethical.

BT595 was well tolerated in all age groups assessed, with no clinically relevant differences in safety parameters between adults or different age groups of pediatric patients. The trial did not raise any safety concerns.

## Conclusions

In summary, the results of the trial demonstrate that BT595 is highly effective as a replacement therapy for pediatric PID patients in reducing the SBI rate and in preventing infections. Pediatric patients achieved similar efficacy and PK outcomes as adults. BT595 is therefore an effective, convenient, well-tolerated, and safe treatment option for children with PID who require prophylactic IVIg treatment.

## Data Availablity

All data and materials were filed in the Trial Master File and the Investigators’ Site Files.

## Data Availability

Not applicable.
